# Targeted metabolomics-based understanding of the sleep disturbances in drug-naïve patients with schizophrenia

**DOI:** 10.1186/s12888-024-05805-0

**Published:** 2024-05-13

**Authors:** Huiming Yan, Gang Li, Xue Zhang, Chuhao Zhang, Meijuan Li, Yuying Qiu, Wei Sun, Yeqing Dong, Shen Li, Jie Li

**Affiliations:** 1 Laboratory of Biological Psychiatry, Institute of Mental Health, Tianjin Anding Hospital, Mental Health Center of Tianjin Medical University, 13 Liulin Rd., Hexi District, Tianjin, 300222 China; 2 Chifeng Anding Hospital, NO.18 Gongger Street, Hongshan District, Chifeng City, 024000 Inner Mongolia Autonomous Region China

**Keywords:** Schizophrenia, Metabolomics, Sleep disturbances, Characteristic metabolites

## Abstract

**Background:**

Sleep disturbances are a common occurrence in patients with schizophrenia, yet the underlying pathogenesis remain poorly understood. Here, we performed a targeted metabolomics-based approach to explore the potential biological mechanisms contributing to sleep disturbances in schizophrenia.

**Methods:**

Plasma samples from 59 drug-naïve patients with schizophrenia and 36 healthy controls were subjected to liquid chromatography-mass spectrometry (LC-MS) targeted metabolomics analysis, allowing for the quantification and profiling of 271 metabolites. Sleep quality and clinical symptoms were assessed using the Pittsburgh Sleep Quality Index (PSQI) and the Positive and Negative Symptom Scale (PANSS), respectively. Partial correlation analysis and orthogonal partial least squares discriminant analysis (OPLS-DA) model were used to identify metabolites specifically associated with sleep disturbances in drug-naïve schizophrenia.

**Results:**

16 characteristic metabolites were observed significantly associated with sleep disturbances in drug-naïve patients with schizophrenia. Furthermore, the glycerophospholipid metabolism (Impact: 0.138, *p*<0.001), the butanoate metabolism (Impact: 0.032, *p*=0.008), and the sphingolipid metabolism (Impact: 0.270, *p*=0.104) were identified as metabolic pathways associated with sleep disturbances in drug-naïve patients with schizophrenia.

**Conclusions:**

Our study identified 16 characteristic metabolites (mainly lipids) and 3 metabolic pathways related to sleep disturbances in drug-naïve schizophrenia. The detection of these distinct metabolites provide valuable insights into the underlying biological mechanisms associated with sleep disturbances in schizophrenia.

**Supplementary Information:**

The online version contains supplementary material available at 10.1186/s12888-024-05805-0.

## Introduction

Sleep disturbances are prevalent and prominent biological symptoms in patients with schizophrenia. Compared to the general population, patients with schizophrenia face a higher risk of experiencing sleep disturbances and related sleep problems [1, 2]. The reported overall prevalence of sleep disturbances among patients with schizophrenia ranges from 63 to 78% [1, 3]. Common sleep disturbances observed in schizophrenia encompass prolonged sleep onset latency, inefficient sleep, frequent nighttime awakenings, and poor overall sleep quality [4, 5]. Importantly, sleep disturbances often precede prodromal symptoms in up to 77% of patients with schizophrenia and persist throughout the course of the disease [6, 7]. Beyond impacting the patients’ quality of life, sleep disturbances also play a role in the pathophysiology and clinical manifestations of schizophrenia [8].

Numerous studies have been undertaken to elucidate the mechanisms underlying sleep disturbances in patients with schizophrenia. Sleep electroencephalogram (EEG) power and sleep-specific oscillatory rhythms provide insights into underlying neuronal activity. Deficiencies in sleep spindle waves and mild impairments in slow waves have been observed in patients with schizophrenia, suggesting their potential as biomarkers for sleep abnormalities in schizophrenia [9–11]. Evidence from a two-sample bidirectional Mendelian randomization study supports a causal relationship between sleep characteristics and schizophrenia [12]. Furthermore, sleep characteristics have been identified as potential therapeutic targets for patients with schizophrenia, with psychotic symptoms, neurocognitive decline, and suicidality showing associations with abnormal sleep architecture [13–15]. Dysregulation of neurotransmitters and pathways related to the biological clock genes may contribute to sleep architecture abnormalities [16]. Recent research has suggested the involvement of the kynurenine pathway and kynurenic acid levels in modulating sleep in schizophrenia [17]. Additionally, a case-control study at Maryland Psychiatric Research Center revealed an association between glutamate levels, poor sleep quality and more pronounced positive symptoms in schizophrenia [18]. Overall, the interplay between sleep and schizophrenia in intricate and multifaceted. Despite advances in understanding the link between sleep disturbances and the pathophysiology of schizophrenia, the biological features and functional significance remain largely elusive. Thus, exploring potential biomarkers could offer valuable insights into the precise mechanisms underlying sleep disturbances in schizophrenia.

Metabolomics studies play a crucial role in capturing minor changes in the body’s functioning, as they offer insights into the chemical expression of metabolites present in biological fluids, cells and tissues [19]. Blood, being a readily available biological fluids, not only reflects the interactions among various factors or pathophysiological processes but also serves as a valuable source for identifying disease-related biomarkers, such as those for diabetes and cancer [20–22]. In the context of human sleep, 328 metabolites have been identified as important [23], with particular enrichment of carnitine, lysophosphatidylcholine (lysoPC), and phosphatidylcholine (PC) in the metabolite category, suggesting a unique role for lipids in mediating the connection between sleep and metabolism [24]. Specifically, amino acid metabolites have been associated with sleep timing, and branched-chain amino acids have been implicated in altered glucose metabolism and sleep disruption. Furthermore, circadian rhythms influence approximately half of the circulating plasma metabolites in sleep-deprived populations, involving amino acid metabolism, lipid metabolism, and neurotransmitter metabolic pathways [25].

However, the relationship between changes in metabolites and sleep disturbances in schizophrenia remains a subject of debate. While primary fatty acid amides have been found to be elevated in schizophrenia, their abnormalities in metabolites do not seem to be associated with changes in the sleep architecture of patients with schizophrenia [26]. It has also been shown that alterations in sleep-wake activity cause metabolic changes in the body [25]. Understanding the metabolic changes in sleep disturbances can facilitate a clearer understanding of the biological mechanisms of sleep disturbances in schizophrenia. Previous studies have explored the unique metabolomics signature of chronic schizophrenia, identifying metabolite changes and potential biomarkers (such as N-acetylaspartate, tryptophan, glutamine, lipids) in chronic schizophrenia [27, 28]. Nonetheless, it is essential to consider the potential influence of antipsychotic drugs, which can also cause metabolite alterations and affect sleep quality.

Although some previous studies have reported metabolite changes in patients with schizophrenia, there is still a lack of consistency in looking at the characteristic metabolites associated with sleep disturbances in schizophrenia. However, antipsychotic drugs can also cause metabolic changes and affect sleep disturbances. Therefore, the present study focuses on drug-naive schizophrenia patients, aiming to identify characteristic metabolites associated with sleep disturbances in schizophrenia patients. To the best of our knowledge, this is the first metabolomic investigation of sleep disturbances in drug-naïve schizophrenia patients. Although the patients did not have identical lifestyles and dietary patterns prior to enrolment, we developed inclusion criteria that were as thorough as possible to allow for a certain degree of similarity in the enrolled patients, providing reliability to the study. Utilizing metabolomics techniques to study characteristic metabolites holds the potential to shed light on the biological mechanisms underlying sleep disturbances in schizophrenia, contributing to a deeper understanding of disease pathology by revealing alterations in metabolic pathways.

## Methods

### Participants

In accordance with a cross-sectional study design, the participants pool was drawn from individuals seeking care at the psychiatric outpatient or emergency department of Tianjin Anding Hospital between November 2017 and August 2022. Eventually, a total of 60 patients (male/female: 17/43) meeting the criteria for schizophrenia or schizophreniform disorder, as outlined by the Clinician Version of the Diagnostic and Statistical Manual of Mental Disorders-fifth edition (DSM-5) Structured Clinical Interview [29], were included. These diagnoses were independently confirmed by 2 experienced psychiatrists. The selected patients adhered to the following inclusion criteria: (1) aged between 18 and 65 years; (2) antipsychotic medication naive or receiving antipsychotic medication for no more than one month, and had received antipsychotic medication for no more than two consecutive weeks prior to enrollment; (3) elementary education or above; (4) the total score of the Positive and Negative Symptom Scale (PANSS) ≥ 60. Exclusion criteria encompassed: (1) presence of sleep-affecting conditions such as obstructive apnea syndrome, narcolepsy, and periodic leg movement disorders; (2) serious physical diseases (neurological disease, acute or chronic somatic disease, especially history of gastric and intestinal surgery); (3) substance abuse (drug or alcohol); (4) pregnancy or nursing; (5) under physical therapy within the past month, such as the electro-convulsive therapy, transcranial magnetic stimulation and electrical stimulation; (6) use of antibiotics or other immune agents, hormones, microecological agents, and probiotics in the past month; (7) presence of evident suicidal tendencies or recent impulsive injuries.

Age- and gender-matched healthy controls (male/female = 16/20) were recruited from the local community and among socially engaged individuals. These controls displayed good physical healthy and lacked any history or familial history of psychiatric diagnoses applicable to the DSM-5. Each subject underwent a comprehensive assessment, including demographic characteristics, physical examinations, and laboratory tests for blood screening. Prior to participating in the study, written informed consent was obtained from each individual or their legal guardian.

### Clinical assessments

Sleep disturbances among all participants were assessed using the Pittsburgh Sleep Quality Index (PSQI) [30]. A clinical cut-off point of 5 was utilized, categorizing a total score of 5 or lower as indicative of “good” sleep, while scores exceeding 5 were indicative of sleep disturbances [30]. Patients with schizophrenia underwent assessment of psychiatric symptoms using the PANSS [31]. A robust inter-rater correlation coefficient exceeding 0.8 was attained for repeated PANSS assessments prior to the study.

### Blood samples collection

Fasting venous blood samples were collected from all participants using EDTA-coated tubes after overnight fasting (≥ 8 h). Each blood sample was divided into 2 aliquots (5 ml each). Subsequently, these aliquots were assayed before noon on the same day to measure parameters, such as high-density lipoprotein cholesterol, low-density lipoprotein cholesterol, fasting plasma glucose, triglycerides, and total cholesterol levels. Afterward, other portions were separated by centrifugation at 1,000r for 15 min at 4℃. The separated plasma samples were stored at − 80 °C for subsequent analysis.

### Metabolomics analysis of plasma samples

All processing and quantitative analysis of plasma samples based on target metabolomics were performed using the P500 kit (Beijing Protein Innovation, BIOCRATES Europe). This kit allows the quantification of 630 metabolites via flow injection analysis coupled with tandem-mass spectrometry (FIA-MS) and liquid chromatography-mass spectrometer (LC-MS) (classification information for the 630 metabolites can be found in Supplementary Table 1). Specifically, a 10µL sample was transferred to a designated location in a 96-well plate and subsequently dried using nitrogen gas. Derivatization was performed using 5% phenyl isothiocyanate, incubated in the dark for 1 h and blown dry by nitrogen. Afterward, 300 µL of extraction solvent was added, mixed at 450 r for 30 min. The filtered extracts were collected through centrifugation and filtration at 600 rpm for 10 min, and diluted 2-fold and 25-fold for LC-MS and FIA mode analysis, respectively. The mass spectrometry analysis of the samples was sequentially performed according to the sample table derived from the MetLMS system. Each sample underwent two separate assessments, with the first involving FIA mode signal acquisition in positive mode (diluted in the Methanol), and the second involving acquisition in LC-MS mode, both in positive and negative modes (0.2% formic acid in water for mobile phase A and in acetonitrile for mobile phase B at 50℃ by using Biocrates® MxP® Quant 500 UHPLC column; Biocrates® Part No.: 22,005). To ensure data quality, sample quality control (QC) procedures were implemented to check the stability of the assay batches. Metabolites with coefficient of variation (CV) exceeding 25% in the QC were filtered out and excluded in the subsequent data analysis. Ultimately, a total of 271 metabolites successfully passed the QC assessment.

### Statistical analysis

A comprehensive overview of the study design and analysis process is outlined in Supplementary Fig. 1. The exported data were subjected to rigorous statistical analyzed employing the R language package. Metabolite concentration values (ng/ml) were consolidated and organized. Participants were segregated into two main groups: GROUP1, encompassing cases and controls; and GROUP2, encompassing cases with sleep disturbances and controls without sleep disturbances. To assess sociodemographic and clinical variables, categorical variables were subjected to chi-square tests, while independent t-tests were applied for continuous variables demonstrating normal distribution. Continuous variables exhibiting non-normal distribution were examined using the Mann-Whitney nonparametric test. Metabolite data were filtered for outliers using Hotelling’s T-squared method.

The construction of classification models involved the utilization of orthogonal partial least squares discriminant analysis (OPLS-DA), employing log10- transformed. The robustness of these models was assessed via a permutation test involving 200-iteration. Significance thresholds of *p* < 0.05 and variable importance in project (VIP) > 1 were used to identify differential metabolites demonstrating differential expression between cases and controls.

Interrelationships between the identified differential metabolites and the 7 factors of PSQI were explored using partial correlation analysis. Further analysis entailed the examination of overlapping differential metabolites across the two primary groups, focusing on those associated with sleep disturbances. Finally, the metabolic pathway analysis was determined by searching the compound databases such as KEGG, HMDB, and PubChem. The software SPSS24.0 was used for our statistical analyses. The construction of OPLS-DA models was facilitated by SIMCA 14.1 software. Pathway analysis outcomes were visualized using the MetaboAnalyst 5.0 software package (https://www.metaboanalyst.ca).

## Results

### Demographic and clinical features

Within the scope of our metabolomic analysis involving 271 metabolites across 96 subjects, one sample was identified as an outlier through the 99% Hotelling’s T-square confidence interval and was subsequently excluded from consideration (pertaining to a drug-naive patient with schizophrenia). Finally, the final study cohort comprised 59 drug-naive patients with schizophrenia (54 drug-naive and 5 drug-free patients) and 36 healthy controls.

 In GROUP1, statistically significant differences in terms of BMI (*t*=-3.443, *p* = 0.001) and marital status (*χ2 =* 23.08, *p* < 0.001) were observed between the two groups. Other demographic and clinical data showed no statistical significances. The PSQI total score of the drug-naïve schizophrenia group was significantly higher than that of controls (7.78 ± 4.39 vs. 4.14 ± 3.60). The demographic and clinical information of subjects is summarized in Table 1.

**Table 1:** Demographic and clinical characteristics of study participants

	Schizophrenia (*N*=59)	Control (*N*=36)	Statistics *t/z/χ* ^*2*^	*P* Value
Gender, M/F	17/42	16/20	2.410	0.121
Age (yrs)	38.07±10.57	38.03±9.66	0.018	0.985
Education (yrs)	12.42±3.45	13.22±3.64	-1.220	0.222
Marital status, Single/Married	43/16	8/28	23.08	<0.001
Smoking, Y/N	8/51	5/31	0.002	0.964
BMI (kg/m^2^)	21.66±3.89	24.42±3.62	-3.443	0.001
Illness duration (mos)	53.61±63.41	NA	NA	NA
PANSS				
P subscore	25.76±4.83	NA	NA	NA
N subscore	20.76±7.17	NA	NA	NA
G subscore	42.54±7.38	NA	NA	NA
Total score	89.07±14.32	NA	NA	NA
PSQI				
Subjective sleep quality	1.27±0.94	0.67±0.79	-3.112	0.002
Sleep latency	1.32±1.11	0.72±0.91	-2.656	0.008
Sleep duration	0.83±1.02	0.78±0.83	-0.059	0.953
Sleep efficiency	0.81±1.09	0.31±0.58	-2.209	0.027
Sleep disturbances	0.81±0.51	0.67±0.48	-1.319	0.187
Sleep medication use	1.48±1.49	0.17±0.61	-4.408	<0.001
Daytime dysfunction	1.37±1.19	0.86±0.90	-1.975	0.048
Total score	7.78±4.39	4.14±3.60	-4.139	<0.001
FPG (mmol/L)	4.89±1.09	5.06±0.33	-2.440	0.015
TC (mmol/L)	4.57±1.05	4.93±0.79	-1.780	0.078
TG (mmol/L)	1.15±0.72	1.29±1.05	-0.307	0.759
HDL-C (mmol/L)	1.42±0.39	1.40±0.33	-0.008	0.994
LDL-C (mmol/L)	2.66±0.78	2.82±0.78	-1.004	0.318

*NA* Not applicable, *PANSS* Positive and Negative Syndrome Scale, *P subscore* Positive symptom subscore, *N subscore* Negative symptom subscore, *G subscore* General psychopathology subscore, *PSQI* Pittsburgh Sleep Quality Index, *BMI* Body mass index, *FPG* Fasting plasma glucose, *TC* Total Cholesterol, *TG* Triglyceride, *HDL-C* High-density lipoprotein cholesterol, *LDL-C* Low-density lipoprotein cholesterol

 In GROUP2, compared with healthy controls, the patients showed a significant increase in total score and each factor of PSQI (all *p* < 0.05). Demographic characteristics of patients with schizophrenia had significant differences in terms of education (*z*=-2.167, *p* = 0.030), marital status (*χ2 =* 15.88, *p* < 0.001), and BMI (*t*=-2.125, *p* = 0.038) (Table 2).

**Table 2:** Demographic and clinical characteristics of Group 2 participants

	Schizophrenia SD (*N*=38)	Control Non-SD (*N*=26)	Statistics *t/z/χ* ^*2*^	*P* Value
Gender, M/F	13/25	10/16	0.121	0.728
Age (yrs)	39.92±10.46	36.27±9.82	1.406	0.165
Education (yrs)	12.53±3.26	14.23±3.02	-2.167	0.030
Marital status，Single/Married	28/10	6/20	15.877	<0.001
Smoking, Y/N	6/32	4/22	0.002	0.965
BMI (kg/m^2^)	21.75±3.84	23.78±3.61	-2.125	0.038
PANSS				
P subscore	25.16±4.51	NA	NA	NA
N subscore	20.55±7.10	NA	NA	NA
G subscore	42.29±6.34	NA	NA	NA
Total score	88.00±11.67	NA	NA	NA
PSQI				
Subjective sleep quality	1.66±0.88	0.31±0.47	-5.462	<0.001
Sleep latency	1.71±1.11	0.35±0.49	-4.731	<0.001
Sleep duration	1.11±1.11	0.46±0.65	-2.248	0.025
Sleep efficiency	1.05±1.16	0.12±0.33	-3.699	<0.001
Sleep disturbances	0.92±0.49	0.54±0.51	-2.846	0.004
Sleep medication use	1.97±1.40	0.04±1.20	-5.218	<0.001
Daytime dysfunction	1.95±1.06	0.62±0.70	-4.592	<0.001
Total score	10.29±3.25	2.38±1.60	-6.772	<0.001

*SD* Sleep disturbances, *NA* Not applicable, *PANSS* Positive and Negative Syndrome Scale, *P subscore* Positive symptom subscore, *N subscore* Negative symptom subscore, *G subscore* General psychopathology subscore, *PSQI* Pittsburgh Sleep Quality Index, *BMI* Body mass index

### Differential metabolites analysis

**Fig. 1:**
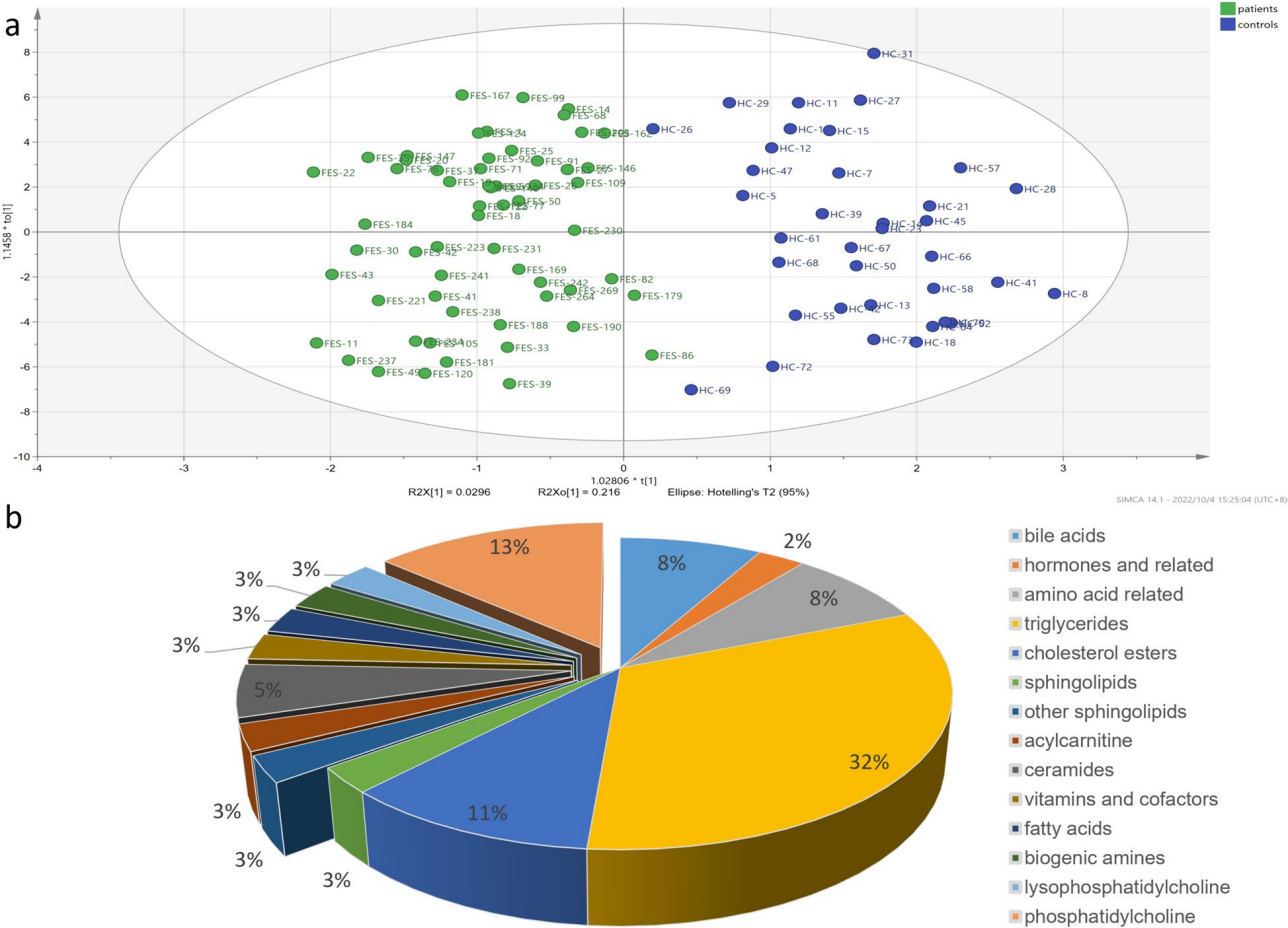
Statistical analysis of normalized datasets of GROUP1. **a** OPLS-DA of cases and controls; (**b**) 37 metabolites with VIP > 1 and *p*  < 0.05 were identified between cases and controls. These metabolites are classified as bile acids, hormones and related, amino acid related, triglycerides, cholesterol esters, sphingolipids, acylcarnitine, ceramides, vitamins and cofactors, fatty acids, biogenic amines, lysophosphatidylcholine, and phosphatidylcholine. The down-regulated metabolites account for 36% on the left and up-regulated metabolites for 64% on the right

**Fig. 2:**
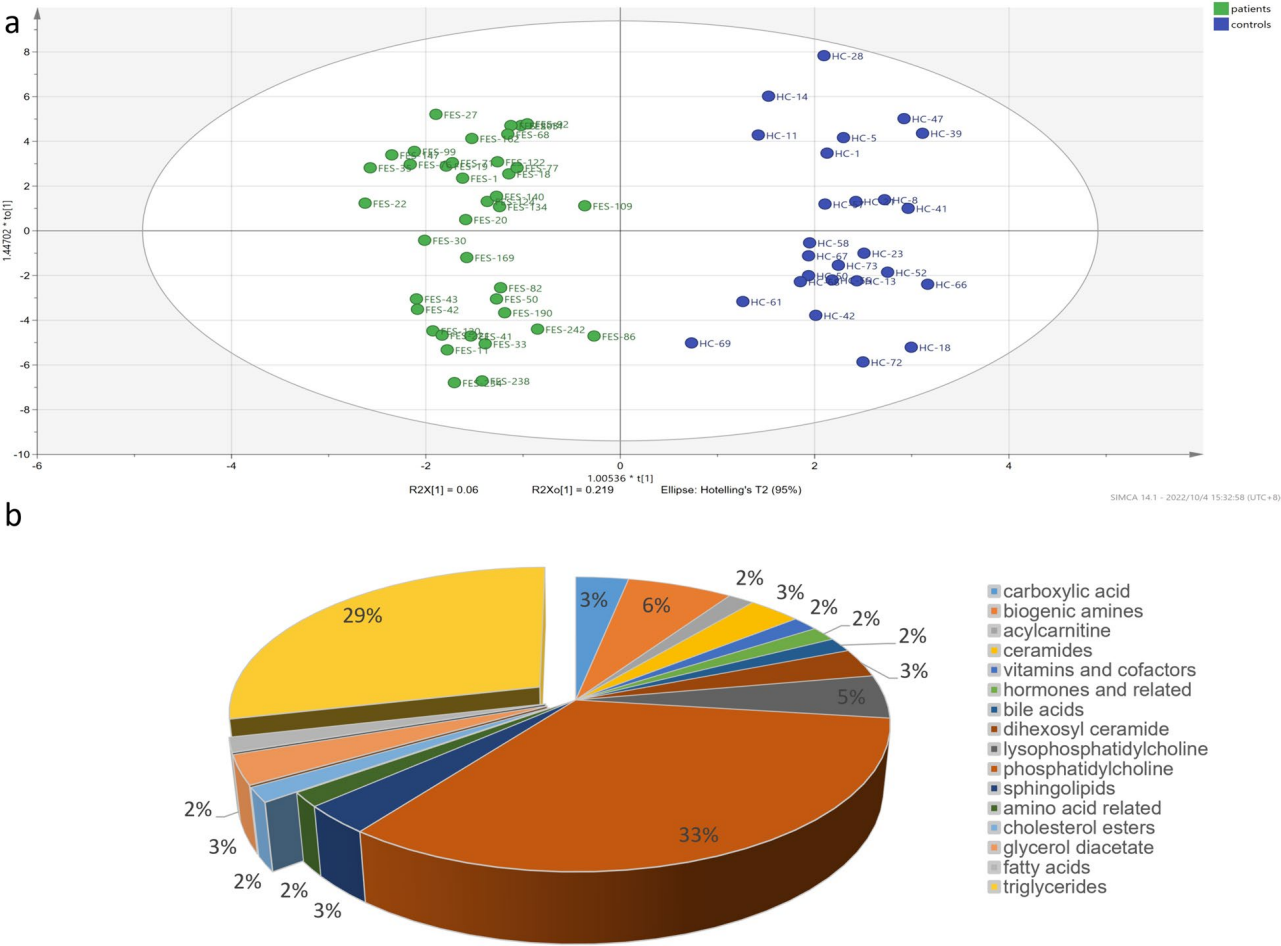
Statistical analysis of normalized datasets of GROUP2.  **a** OPLS-DA of cases with sleep disturbances and controls without sleep disturbances; (**b**) 63 metabolites with VIP > 1 and *p*  < 0.05 were identified between cases with sleep disturbances and controls without sleep disturbances. These metabolites are classified as carboxylic acid, biogenic amines, acylcarnitine, ceramides, vitamins and cofactors, hormones and related, bile acids, dihexosyl ceramide, lysophosphatidylcholine, phosphatidylcholine, sphingolipids, amino acid related, cholesterol esters, glycerol diacetate, fatty acids, and triglycerides. The up-regulated metabolites account for 35% on the left and down-regulated metabolites for 65% on the right

The application of the OPLS-DA model effectively demonstrated a clear demarcation between the two groups, signifying substantial metabolic disparities between patients and healthy controls. In OPLS-DA of GROUP1, the overfitting R2Y of the model was 0.822, and the predictive power Q2 was 0.522. Out of the pool of 271 metabolites, a subset of 51 displayed differential expression with *p*-values below 0.05, and 88 metabolites demonstrated a VIP score exceeding 1. Finally, 37 differential metabolites with *p* < 0.05 and VIP > 1 were screened (Fig. 1). In addition, for GROUP2, which encompassed 38 patients with sleep disturbances and 26 controls without such sleep disturbances, the outlined analytical procedure was independently conducted. The OPLS-DA model of GROUP2 has an R2Y of 0.92 and a Q2 of 0.564. A total of 63 differential metabolites were identified out of 271 metabolites according to *p* < 0.05 and VIP > 1 (Fig. 2).

### Associations between characteristic metabolites and sleep disturbances

Given the potential impact of factors like body mass index (BMI) on metabolite, partial correlation analysis was conducted to mitigate the influence of confounding factors according to the aforementioned disparities. Following the identification of overlapping cross-differential metabolites in both GROUP1 and GROUP2, 16 metabolites emerged as definitive plasma characteristic metabolites associated with sleep disturbances in schizophrenia. Correlation analysis showed that sleep disturbances were positively correlated with the increase of TG (18:1_36:1), TG (22:4_32:0), TG (22:5_32:1) and CE (16:1) (all *p* < 0.05). In addition, the decrease of C2, Cer (d18:1/22:0), Cer (d18:1/24:0), Choline, GABA, lysoPC a C18:0, PC aa C36:0, PC aa C36:2, PC ae C34:0, PC ae C38:2, PC ae C40:1, and SM C24:0 were negatively associated with sleep disturbances (all *p’s* < 0.05) (Table 3).

**Table 3.**
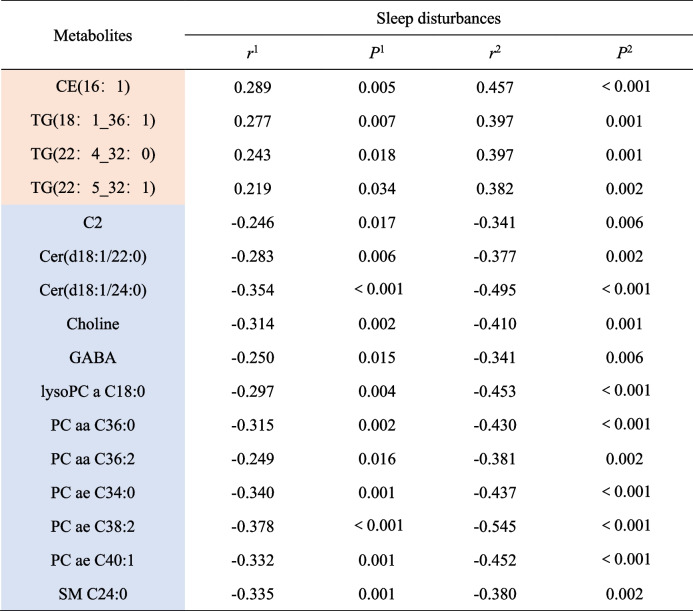
Characteristic metabolites associated with sleep disturbances in schizophrenia

*r* 1 and P 1indicate the partial correlation coefficients and *P*-values in CROUP1. *r* 2 and P 2 indicate the partial correlation coefficients and P-values in CROUP2. Pink coloured markers are up-regulated metabolite concentration values and blue coloured markers are down-regulated metabolite concentration values

### Metabolic pathways of sleep disturbances in schizophrenia

**Fig. 3:**
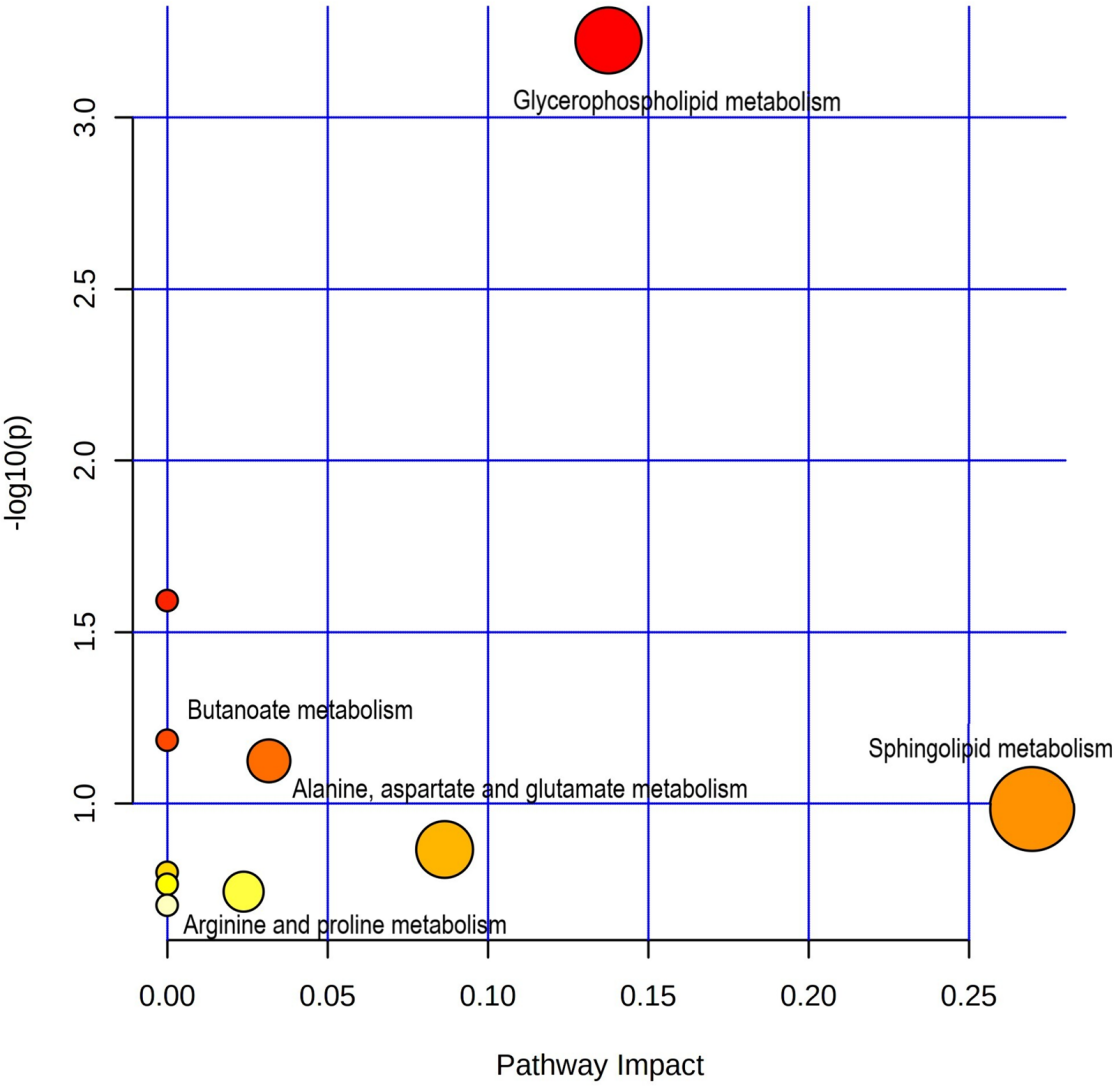
Result of pathway analysis. The vertical -LOG (*p*) value is derived from pathway enrichment analysis and horizontal axes Pathway Impact value is derived from pathway topology analysis. The larger the -LOG (p) the more pronounced the difference, the larger the Pathway Impact value the greater the role of the metabolite in the pathway

Drawing upon the aforementioned observations, 10 important metabolic pathways for sleep disturbances in drug-naïve schizophrenia were identified by pathway analysis (Fig. 3). The more significant the pathway impaction, the greater its effect on the metabolic pathway of sleep disturbances in schizophrenia. As shown in Fig. 3, these crucial pathways encompass glycerophospholipid metabolism (Impact: 0.138, *p* < 0.001), butanoate metabolism pathways (Impact: 0.032, *p* = 0.008), and sphingolipid metabolism (Impact: 0.270, *p* = 0.104).

## Discussion

This study marks the first attempt to quantitatively assess the metabolite profile associated with sleep disturbances in schizophrenia through a targeted metabolomics approach. The results unveiled a conspicuous divergence in metabolomic profiles between schizophrenia patients and controls, and culminating in the identification of 16 characteristic metabolites associated with schizophrenia sleep disturbances, primarily constituting lipid species. In addition, the metabolic pathways most profoundly impacted in individuals with schizophrenia-related sleep disturbances encompassed glycerophospholipid metabolism, butanoate metabolism and sphingolipid metabolism.

Several characteristic metabolites of schizophrenia emerged, with a majority belonging to the lipid category, including triglycerides, PC, lysoPC, acylcarnitines and sphingolipids. Our findings align harmoniously with previous reports indicating analogous divergences in metabolite profiles in patients with schizophrenia using LC-MS techniques [32–34]. Lipid-related metabolites are an important class of cellular and intracellular compounds, and alterations in the lipid profile may occur early in the development of schizophrenia disease [35]. Of particular note, the observed decline in PC and lysoPC levels in schizophrenia patients mirrors patterns observed in the glycerophospholipid metabolism. This corroborates findings from previous research spotlighting diminished glycerophospholipid metabolism within the prefrontal cortex of individuals with schizophrenia [36]. Acylcarnitines, exemplified by C2, are important regulators of lipid metabolism and play an important role in countering bioenergetic metabolic dysfunction in schizophrenia [37]. Collectively, this underscores the substantive engagement of lipid metabolites in the pathological dynamics of schizophrenia, highlighting the central role of lipid models in comprehending the intricacies of this condition.

In the present study, patients with sleep disturbances exhibited a notable down-regulation in the majority of metabolite levels, with the exception of an up-regulation in triglycerides and cholesterol ester levels. Similar results were supported in human plasma samples of sleep deprivation and sleep restriction [38, 39]. Similar alterations in lipid metabolites, such as triglycerides, PC, and lysoPC, have been observed in women with poorer sleep quality [40], . Investigations into human sleep deprivation have also unveiled shifts in over a third of lipid metabolites, including a decline in choline plasmalogen levels and a rise in several triacylglycerides and PC species (lysoPC 16:1, PC 34:3, PC 36:3, PC 38:3, and PC 40:7, etc.) [39, 41]. Intriguingly, temporary sleep supplementation appeared incapable of restoring reduced acylcarnitine levels [38].

The effect of metabolites on sleep dynamics may be related to ABC transporters, which play pivotal roles in lipid homeostasis, phospholipid metabolism, and sphingolipid metabolism [42]. Specifically, sleep deprivation (no more than 5 h of sleep per night) have been connected to the down-regulation of ABC transporter proteins [43], consequently heightening the vulnerability of sleep deprivation-related diseases. Notable shifts in sphingomyelin (SM 43:2, SM d33:2) and sphingolipids ceramide (Cer 40:2, Cer d41:2) have also been noted under conditions of insufficient sleep [42]. It has been proposed that the concentrations of human ceramides and sphingomyelin are negatively correlated with sleep energy expenditure [44]. The putative cellular mechanism is an increase in sphingolipid-induced uncoupling, which decreases mitochondrial respiratory activity [45, 46]. Comparable perturbations have also been observed in brain tissue metabolite profiles in animal models of sleep deprivation [47]. Interestingly, both blood and brain metabolic profiles in rats showed a tendency to recover after the administration of interventions [48]. A rat model simulating chronic sleep restriction showed impaired circulating fatty acids, including triglycerides, choline, and PC, following sleep deprivation, accompanied by reduced glucose tolerance and impaired insulin secretion [49]. These studies underscore the widespread metabolic shifts that transpire in sleep disturbances.

To be noted, our study highlights the negative association of C2 and GABA with sleep disturbances in schizophrenia. C2 is a metabolite of fatty acids, and supplementation with C2 increases the production of releasable glutamate [50]. Glutamate is an excitatory neurotransmitter and a precursor for the synthesis of inhibitory GABA. Glutamate could mediate rapid eye movement sleep and is associated with the initiation and maintenance of the sleep/wake cycles [51]. Meanwhile, GABAergic neurons in the brain are key factors to promote sleep [52]. Our findings accentuate the depletion of C2 and GABA during sleep disturbances, underscoring their regulatory influence on the normative sleep architecture.

Through metabolic pathway analysis, our study revealed that glycerophospholipid metabolism and butanoate metabolism exhibited the most significant alterations associated with sleep disturbances between patients and controls. Supplementary to these, sphingolipid metabolism and other pathways were also notably affected. Alterations in glycerophospholipid metabolism are associated with the pathophysiological mechanisms of schizophrenia [36, 53]. Furthermore, the lipid content and enzyme expression activity of glycerophospholipid metabolism in the nervous system and neurons are regulated to the highest degree by the circadian clocks among the various metabolisms. The circadian-related genes (e.g., *per1*) are involved in the synthesis of cells in glycerophospholipid metabolism [54]. Furthermore, sphingolipid metabolism appears to serve as a potential nexus linking short sleep duration and the risk of neurodegenerative diseases. Phospholipid metabolism (lysoPC- [18:3] and PC- [40:5]) has been implicated in the inflammatory and metabolic diseases associated with sleep deprivation [42, 55–57]. In addition, the prominence of butanoate metabolism toward gut microbiome involvement. Notably, previous research has indicated functional impairment in the butanoate metabolism component of the gut microbiome in individuals with poor sleep quality group compared to healthy subjects [58]. Future integration of the gut microbiome datasets could potentially unveil metabolite alterations.

Our study has several limitations to acknowledge. First, the relatively small sample size and uneven gender distribution might introduce biases in the statistical analysis. Furthermore, differences in BMI between the two groups could potentially confound the results of the metabolomic analysis, as BMI variations have been associated with changes in metabolite categories such as lipids, sex steroids, amino acids, and acylcarnitine [59]. Second, crucial lifestyle and physical factors influencing the metabolomic outcomes, including physical comorbidities, alcohol drinking, dietary habits, work and rest patterns, and physical activity, were not collected. Third, the reliance on the PSQI, which reflects habitual sleep patterns, could be susceptible to recall biases. Integrating polysomnographic efforts may better provide the potential to assess sleep disturbances. Fourth, our study’s cross-sectional design hindered the ability to track longitudinal changes in sleep disturbances. Further studies should aim to address these limitations by rigorously screening for BMI differences between groups and striving to balance BMI distributions to mitigate potential confounding effects. Additionally, ongoing recruitment efforts and longitudinal studies are essential to elucidate the impact of characteristic metabolites on sleep disturbances in schizophrenia. This will provide more insight into the characteristic metabolites associated with sleep disturbances in schizophrenia.

In addition, this study has several strengths. First, we used targeted metabolomics analysis to assess the levels of characteristic metabolites in the plasma of schizophrenia patients and healthy controls. Compared to untargeted metabolomics, targeted metabolomics has excellent selectivity and sensitivity while providing data. In addition, plasma metabolomics allows for a deeper understanding of the relationship between small molecules in the blood and body metabolism. We searched for several of the most affected metabolic pathways through the discovery of characteristic metabolites in patients with schizophrenia sleep disturbances, suggesting that it could provide the basis for future longitudinal studies to better optimise early detection or intervention of sleep disturbances in schizophrenia patients.

## Conclusion

In conclusion, our study employing targeted metabolomics has identified 16 meaningful characteristic metabolites associated with sleep disturbances in schizophrenia. These metabolites show substantial enrichment within glycerophospholipid metabolism, butanoate metabolism and sphingolipid metabolism pathways. The identified characteristic metabolites, primarily lipid-based, hold promise as potential biomarkers for detecting sleep disturbances in schizophrenia patients. The insights garnered from metabolomics investigations could offer novel avenues for delving into the underlying biological mechanisms of sleep disturbances in drug-naïve individuals grappling with schizophrenia.

### Supplementary Information

Supplementary Material 1.

## Data Availability

The datasets generated and analyzed during the current study are not publicly available to protect the privacy of research participants but are available from the corresponding author on reasonable request.
