# Supplementary material for: Targeted metabolomics-based understanding of the sleep disturbances in drug-naïve patients with schizophrenia
Source: BMC Psychiatry. 2024 May 13;24:355. doi: 10.1186/s12888-024-05805-0 (PMC11089724; doi:10.1186/s12888-024-05805-0)
Supplement: Supplementary file 1 — Supplementary Material 1. [file 12888_2024_5805_MOESM1_ESM.docx]

Supplementary Figure.1 Research methodolody design chart


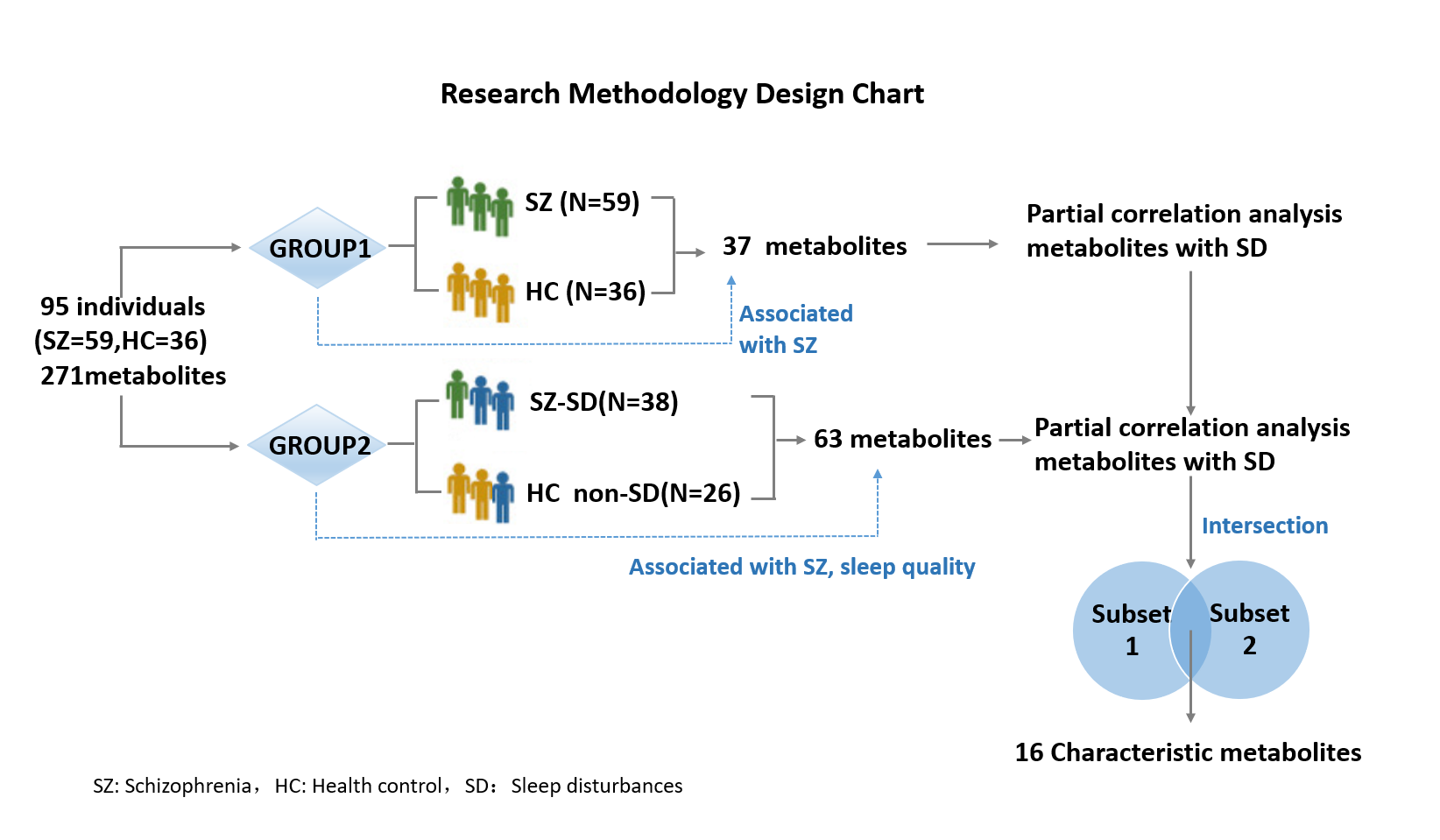


Supplementary Table.1 630 metabolites classification information

| Target classification | Total number |
| --- | --- |
| Acylcarnitine | 40 |
| Alkaloids | 1 |
| Amine oxide | 1 |
| Amino acids | 20 |
| Amino acid related | 30 |
| Bile acids | 14 |
| Biogenic amines | 9 |
| Carbohydrates and related | 1 |
| Cholesterol esters | 22 |
| Carboxylic acid | 7 |
| Glycerol diacetate | 44 |
| Dihydroceramide | 8 |
| Cresol | 1 |
| Fatty acids | 12 |
| Hormones and related | 4 |
| Indole and its derivatives | 4 |
| Ribonucleases and related | 2 |
| lysophosphatidylcholine | 14 |
| Phosphatidylcholine | 76 |
| Sphingolipids | 15 |
| Ceramides | 28 |
| Ethyl ceramide | 19 |
| Glycerol triglycerides | 242 |
| Vitamins and cofactors | 1 |
| Dihexosyl ceramide | 9 |
| Trihexyl ceramide | 6 |

Target classification is a categorisation of the targets contained in the kit based on the nature of the metabolite. The total number is the number of targets covered by the kit under that classification.
